# Impact of Anesthesia With Propofol on Epileptic Discharges Recorded by Stereo‐Electroencephalography in Pediatric Epilepsy Surgery

**DOI:** 10.1002/cns.70767

**Published:** 2026-01-27

**Authors:** Chang Liu, Ping Ding, Tong Zhao, Tinghong Liu, Shijie Wu, Yuxiang Yan, Liu Yuan, Liwei Zhang, Shuli Liang

**Affiliations:** ^1^ Department of Functional Neurosurgery, National Center for Children's Health of China Beijing Children's Hospital, Capital Medical University Beijing China; ^2^ Key Laboratory of Major Diseases in Children Ministry of Education Beijing China; ^3^ Department of Neurosurgery, Renji Hospital, School of Medicine Shanghai Jiao Tong University Shanghai China; ^4^ Gnosis Neurodynamics Co. Ltd Beijing China

**Keywords:** anesthesia, epilepsy surgery, high‐frequency oscillation, intra‐operative electroencephalography, power spectral density

## Abstract

**Objective:**

This study aimed to evaluate the influence of anesthesia with propofol on epileptic discharges recorded by stereo‐electroencephalography (SEEG) during pediatric epilepsy surgery.

**Methods:**

We enrolled 30 pediatric patients (aged 2–14 years) undergoing epilepsy surgery between June 2022 and November 2023. SEEG recordings were obtained under different states of consciousness: awake, sleep, and anesthesia. We analyzed the power spectral density (PSD) across frequency bands and quantified the number of interictal spikes and high‐frequency oscillations (HFO) including ripples and fast ripples (FRs).

**Results:**

There was a significant reduction in the number of spikes, particularly within the seizure onset zone (SOZ), while ripples and FRs increased under general anesthesia, but the extent of the increase did not differ between the SOZ and other detected brain regions (NSOZ). Analysis of PSD showed increased energy during anesthesia, particularly in high‐frequency bands. Meanwhile, no statistical differences were observed in energy changes between the SOZ and NSOZ when compared to wakefulness or sleep. HFOs emerged as more robust and consistent interictal biomarkers for localizing the SOZ under propofol anesthesia, particularly in the frontal cortex and cingulate cortex, and were not influenced by age.

**Conclusions:**

General anesthesia with propofol significantly affects the number and frequency of epileptic discharges, particularly by reducing spikes while increasing HFOs. Notably, HFOs—particularly in the frontal and cingulate cortices—emerged as reliable biomarkers for delineating the SOZ and guiding surgical resections. Furthermore, no significant age‐related variability was identified, supporting the applicability of intra‐operative electroencephalography in pediatric epilepsy surgery.

## Graphical abstract

The number of spikes decreased under anesthesia, particularly within the SOZ, while HFOs increased under anesthesia. However, the extent of the increase did not differ between the SOZ and NSOZ. The number of HFOs appeared to be a more reliable interictal biomarker in ioEEG, particularly in the frontal cortex and cingulate cortex, and was not influenced by age.

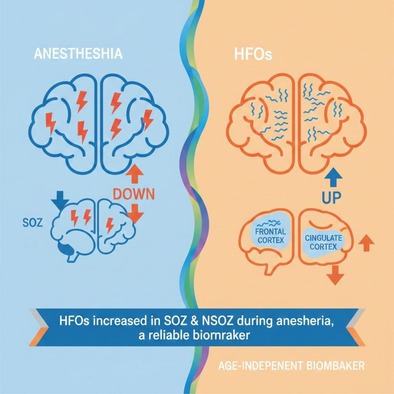

**Abbreviations**
HFO: high‐frequency oscillation
Io‐EEG: intra‐operative electroencephalography
PSD: power spectral densit
SEEG: stereo‐electroencephalography
SOZ: seizure onset zone

## 1 Introduction

Epilepsy surgery is the only potentially curative treatment for patients with drug‐resistant epilepsy, offering a realistic prospect of seizure freedom [1, 2]. The primary objective of surgical intervention is to remove or disconnect the entire epileptogenic zone, which is defined as the cortical region essential for seizure generation [3]. Meanwhile, the seizure onset zone (SOZ) generates all the usual seizures and is the only one we can directly measure before surgery [3]. Intra‐operative electroencephalography (ioEEG) was first introduced by Penfield and Jasper in the late 1930s at the Montreal Neurological Institute to facilitate the intraoperative identification of epileptic brain tissue [4]. This technique records epileptiform activity directly from the cortex during epilepsy surgery, aiding in the delineation of the epileptogenic focus. This so‐called “tailoring” approach can significantly influence surgical decision‐making [5]. Additionally, ioEEG offers several advantages over chronic intracranial EEG monitoring, including the elimination of patient cooperation requirements, direct access to deep brain structures via the surgical cavity, and the ability to evaluate post‐resection remnants of the epileptogenic cortex [6].

However, the role of ioEEG in guiding epilepsy surgery remains a topic of debate. The limitations of ioEEG can be summarized into three main aspects: (1) its reliance on interictal data within the spatiotemporal constraints of the surgical setting, (2) the limited duration of monitoring compared to chronic intracranial EEG, and (3) its high susceptibility to the effects of anesthetic agents, with pharmacological activation also being a potential confounder [7]. With the advancement of research on high‐frequency oscillations (HFOs) in recent years, HFOs have emerged as a promising interictal biomarker capable of addressing the first two limitations of ioEEG. Specifically, intraoperative monitoring of HFOs enables the detection of these events within a relatively short recording duration, allowing for the delineation of the SOZ. Therefore, a key challenge in enhancing the accuracy and clinical utility of ioEEG in defining the surgical resection extent lies in determining whether HFOs recorded via ioEEG are influenced by anesthesia.

Moreover, there is a scarcity of data and considerable heterogeneity regarding the efficacy and reliability of ioEEG in resective epilepsy surgery. Multiple factors, including epileptic pathology [8, 9], temporal versus extra‐temporal epilepsy [10], and MRI‐positive versus MRI‐negative epilepsy [11], have been considered, but the findings remain variable. To the best of our knowledge, no studies have specifically investigated the value of ioEEG in different cortical regions or examined age‐related variability in children. In this study, to further explore the effects of anesthetic agents on ioEEG, we analyzed interictal epileptiform discharges, including spikes, ripples, and FRs, using stereo‐electroencephalography (SEEG) across three different consciousness states, including awake, sleep, and anesthesia. Additionally, we aimed to assess the extent to which anesthesia influences HFOs across different cortical regions and age groups, thereby providing further evidence to support the individualized application of ioEEG.

## 2 Methods

### 2.1 Patients and the Definition of Cortex Division

In this study, cortical division was primarily based on the characteristics of clinical ioEEG application and the distribution of SEEG electrodes in this research. Specifically, the brain is divided into five major areas including the temporal lobe, frontal lobe, insular lobe, central area (precentral gyrus and postcentral gyrus), and posterior area (parietal lobe and occipital lobe). Furthermore, these five brain areas were further subdivided into 10 distinct cortical regions: the mesial temporal cortex, lateral temporal cortex, mesial frontal cortex, lateral frontal cortex, orbital frontal cortex, insular cortex, insular operculum cortex, Rolandic cortex, posterior cortex, and cingulate cortex.

This study included patients who were admitted to the hospital between June 2022 and November 2023. The inclusion criteria were as follows:

1. Children with diagnosis of focal epilepsy and requiring evaluation with SEEG.
2. Patients aged between 2 and 14 years.
3. Subjects with SEEG data recorded under standardized general anesthesia for at least 20 min.
4. Subjects with interictal EEG data recorded by SEEG during sleep and awake.
5. Patients with confirmed SOZ based on ictal SEEG data.

The exclusion criteria were:

1. Subjects whose bispectral index value was below 40 or above 60 for more than 5 min during the recording of anesthetic‐state data,
2. Patients with coverage of fewer than five of the aforementioned cortical regions by SEEG electrodes.

### 2.2 Anesthesia

**FIGURE 1:**
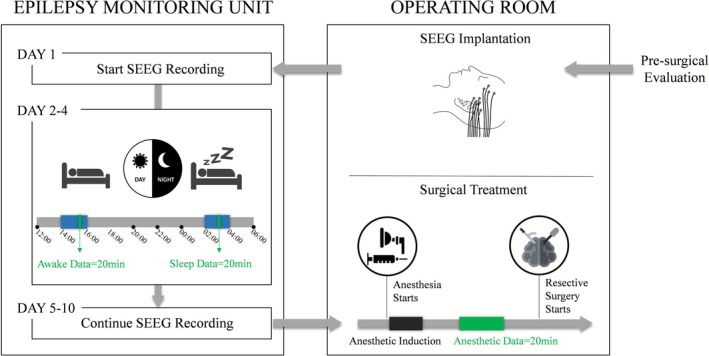
Flowchart of the study. SEEG data were collected from both the operating room and the epilepsy monitoring unit. Green squares represent the acquired data.

Before anesthesia induction, invasive blood pressure, heart rate, electrocardiogram, respiratory activity, oxygen saturation, and bispectral index were continuously monitored. General anesthesia was induced with intravenous propofol (3 mg/kg), sufentanil (0.2–0.3 μg/kg), and rocuronium (0.6 mg/kg). After induction, propofol was administered via continuous intravenous infusion and titrated to a maintenance rate of 9–16 mg/kg·h, while remifentanil was infused at 0.1 μg/kg·min. SEEG recording began after induction and during the steady‐state maintenance phase of anesthesia, and no recordings were obtained during induction or emergence. Depth of anesthesia was monitored using bispectral index, which was maintained between 40 and 60 throughout SEEG acquisition (Figure 1).

### 2.3 SEEG Recording

SEEG recordings in this study included awake data, sleep data, and anesthetic‐state data. All recordings were acquired using a Natus Xltek video‐EEG system with a sampling rate of 2000 Hz. Awake SEEG data were recorded for 20 min between 14:00 and 16:00 on the second to fourth day of post‐electrode implantation under eyes‐closed, undisturbed conditions. Sleep SEEG data were collected for 20 min during the slow‐wave sleep phase, recorded between 02:00 and 04:00, ensuring a disturbance‐free environment. Anesthetic‐state SEEG data were obtained for 20 min following anesthesia induction, under similarly undisturbed conditions (Figure 1).

### 2.4 Signal Preprocessing

Data preprocessing was performed to remove unwanted signal components. Raw signals were first bandpass‐filtered between 0.1 and 500 Hz to attenuate low‐frequency drifts and high‐frequency noise, followed by a notch filter (50 Hz and its harmonics) to suppress line interference. Data were then re‐referenced using a bipolar montage, subtracting adjacent electrode contacts to minimize volume‐conducted noise and enhance local field potentials. The preprocessed SEEG data were then used for subsequent calculations of band energy and detection of abnormal discharges.

### 2.5 Band Energy Calculation

We utilized the Welch method to calculate the energy of different frequency bands. The Welch method is a spectrum estimation technique based on segment averaging. It involves dividing the signal into multiple overlapping segments, performing a Fourier transform on each segment, and averaging the results to reduce noise interference. The power spectral density (PSD) is calculated using the following formula:

$$S_{x}\left(f\right)=\frac{1}{N_{w}}\sum_{k=0}^{N_{w}-1}{\left|F\left[w\left(t\right)\cdot x\left(t-k\Delta t\right)\right]\right|}^{2}$$

where $S_{x}\left(f\right)$ represents the PSD, $x\left(t\right)$ is the original signal, F denotes the Fourier transform, $w\left(t\right)$ is the window function, Δt is the offset of the sliding window, and $N_{w}$ is the number of segments. A time window of 1‐s with a 0.5‐s overlapping sliding window was used for signal segmentation. A Hamming window was applied to minimize spectral leakage. For the frequency bands of interest (delta: 1–4 Hz, theta: 4–8 Hz, alpha: 8–13 Hz, beta: 13–30 Hz, gamma: 30–50 Hz, high gamma: 50–80 Hz, ripple: 80–250 Hz, FR: 250–500 Hz), the mean power within each band was calculated as the band energy. For different cortical regions, the signals from each electrode within the region are first averaged to obtain the average signal, which is then considered as the EEG signal for the respective cortical region. Subsequently, the energy in each frequency band is calculated using the aforementioned method. The logarithm of the energy was taken for visualization purposes. When comparing SOZ with NSOZ in the patient‐level analysis, the energy of each electrode was first calculated. Then, the mean energy within the electrodes located in the SOZ and NSOZ was computed for SOZ/NSOZ analysis, while the mean energy across all electrodes of the patient was calculated for age‐group analysis.

### 2.6 Interictal Epileptic Discharges Detection

#### 2.6.1 Spike Detection

Spikes are more prominent in higher frequency bands. Thus, we first applied a high‐pass filter at 5.3 Hz to the SEEG data. After filtering, peak points were identified, and their amplitudes were ranked in descending order. Peak points were selected based on a proportion of the data duration as candidate spikes. Each candidate spike was evaluated based on features extracted, including: peak amplitude, the ratio of the peak amplitude to the median amplitude within ±1 s, and the slopes of the waveform's rising and falling edges. Thresholds for these features were determined based on prior clinical knowledge. A waveform was classified as a spike if all extracted features met the respective thresholds. For the detected spikes, spatial constraints were applied. Spikes within the same channel exhibit stereotypical, repetitive waveform patterns. Therefore, similarity assessments were conducted for waveforms detected in the same channel. Candidate waveforms from the same channel were averaged to create a template, and only those waveforms with high similarity to the template were retained.

#### 2.6.2 High Frequency Oscillations Detection

For the detection of HFOs including ripples and FRs, we applied band‐pass filters with frequency ranges of 80–250 Hz and 250–500 Hz, respectively, using a fifth‐order Butterworth filter on the SEEG data. Subsequently, the Hilbert transform was employed to extract the signal's energy envelope. A detection threshold was set for the energy envelope as follows:

$$\text{Thres}h_{c}=\max\;\left(2*T_{c},2*T_{g}\right)$$

Here, c represents the contact point, and $T_{c}$ and $T_{g}$ refer to the median of the energy envelope for contact c and the median of the energy envelope across all contacts, respectively. Then, detection segments with intervals shorter than 20 ms were merged, and a window of 50–200 ms was selected. Additionally, the average energy within the window was required to be at least 1.5 times the surrounding baseline energy. HFOs often exhibit a tendency for synchronization; therefore, synchronization constraints were applied to the detection results. All detection results were mapped onto a unified timeline, and a threshold filter was applied to exclude isolated noise events. For different cortical regions, abnormal discharges are first detected at each electrode within the region. The mean number of abnormal discharges detected across all electrodes in the region is then considered as the abnormal discharge count for that cortical region.

### 2.7 Statistical Analysis

In the patient‐level analysis, the number of epileptic discharges in each electrode was first detected. Then, the mean number of discharges within the electrodes located in the SOZ/NSOZ regions was computed for SOZ/NSOZ analysis, while the mean number across all electrodes of the patient was calculated for age‐group analysis.

All data are presented as mean ± standard deviation (SD). Paired *t*‐tests were employed to compare both band energy and interictal discharges counts (including spikes, ripples, and FRs) between different states of consciousness, as well as between SOZ and NSOZ. An independent *t*‐test was used in the statistical analysis between age groups. A *p*‐value of < 0.05 was considered statistically significant. Statistical analyses were conducted using the SciPy package in Python, with Python version 3.7.5 and SciPy version 1.7.3.

## 3 Results

### 3.1 Patients

Thirty patients (18 males) were enrolled in this study, with a mean age of 7.3 ± 3.5 years (range: 2–14 years) at the time of surgery. In total, 259 SEEG electrodes were implanted, with a median of 8.6 electrodes per patient (range: 6–12 electrodes). Twenty‐three patients had unilateral implantation (left = 11), while the remaining seven patients underwent bilateral implantation. The patients were divided into three age groups: 11 patients in the 2–5 years group, 10 in the 6–9 years group, and 9 in the 10–14 years group. Postoperative pathological diagnoses included focal cortical dysplasia (*n* = 25), tuberous sclerosis complex (*n* = 3), Sturge–Weber Syndrome (*n* = 1), and encephalomalacia (*n* = 1).

### 3.2 Power Spectral Density

**FIGURE 2:**
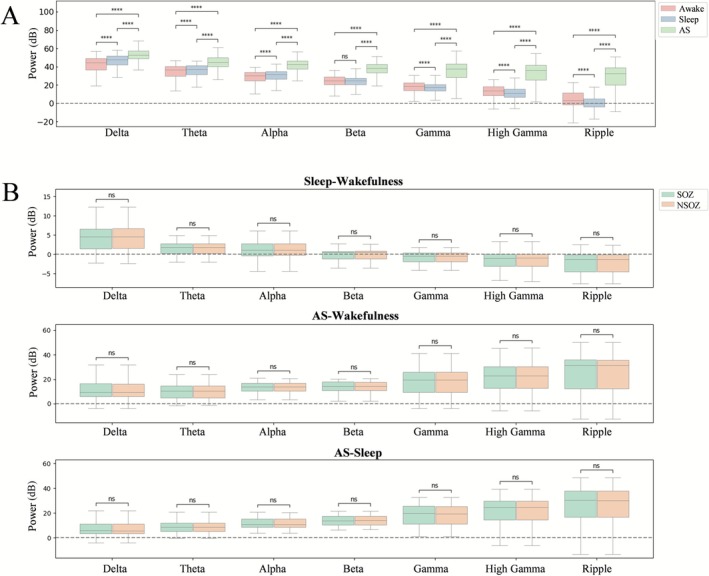
Results of PSD analysis. (A) PSD analysis across wakefulness, sleep, and anesthetic states on different bands. (B) Changes in power associated with shifts in consciousness, calculated by comparing differences in power across the three states within each frequency band. p1, AS, anesthetic‐state; ns, nonsignificant.

PSD analysis across different states of consciousness revealed significant differences in power within the delta, theta, alpha, gamma, high gamma, and ripple frequency bands among wakefulness, sleep, and anesthesia. In the beta frequency band, power during anesthesia differed significantly from both wakefulness and sleep (Figure 2A, Table S1).

When examining the energy changes associated with shifts in consciousness by calculating the difference in power across the three states within each frequency band, no significant changes were observed between wakefulness and sleep in either the SOZ or NSOZ. Moreover, anesthesia‐induced changes in power were not statistically different between the SOZ and NSOZ across all frequency bands, regardless of whether the comparison was made against wakefulness or sleep (Figure 2B, Table S2).

### 3.3 Epileptic Discharges

**FIGURE 3:**
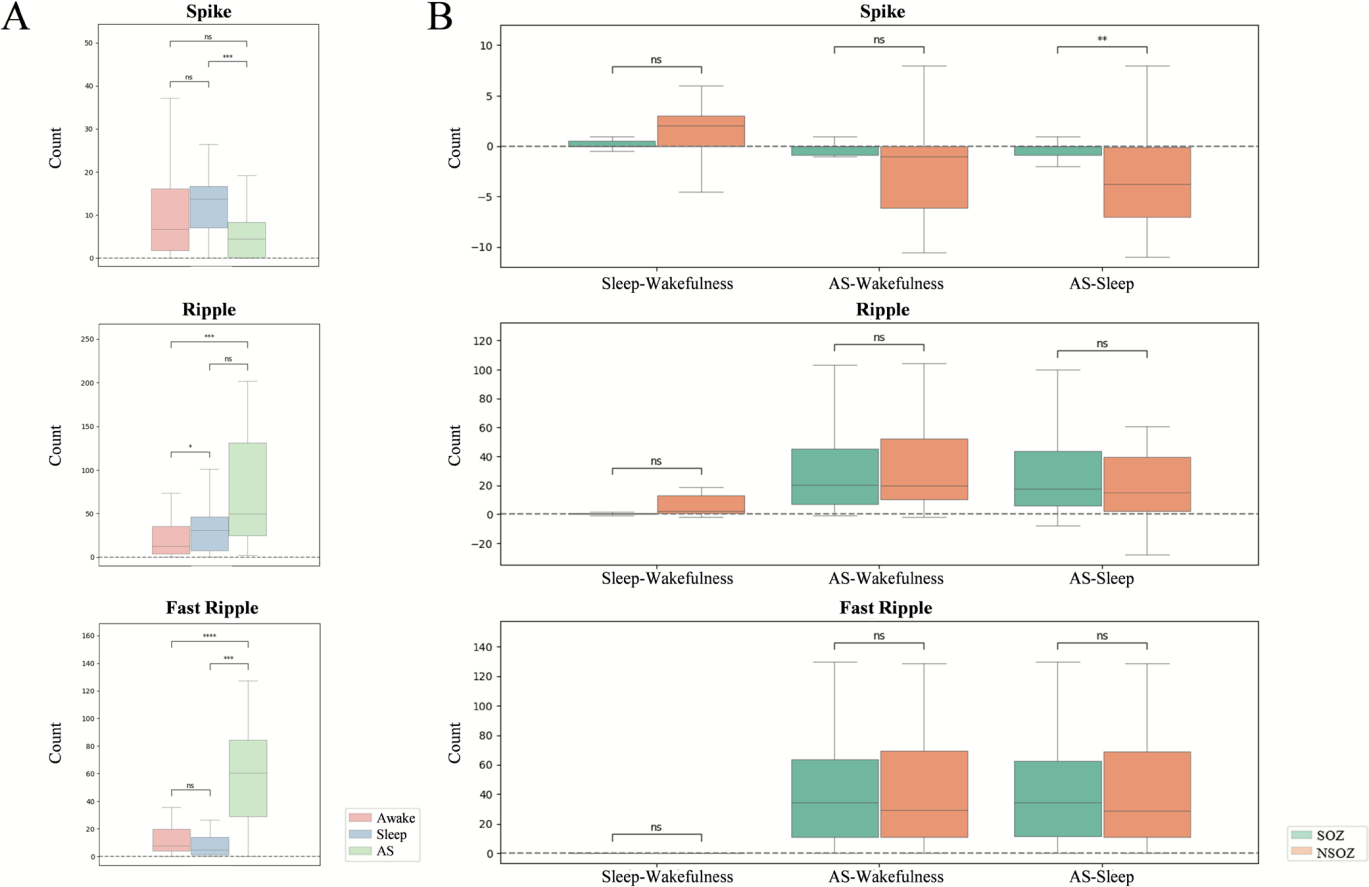
Quantification of interictal epileptic discharges. (A) The distribution of three types of interictal epileptiform discharges across wakefulness, sleep, and anesthetic‐state. (B) Changes in interictal epileptiform discharges between the SOZ and NSOZ across different states of consciousness. ***p < 0.001, ****p < 0.0001, AS, anesthetic‐state; ns, nonsignificant.

Comparing the changes in three types of interictal epileptiform discharges across wakefulness, sleep, and anesthetic‐state, the results showed: (1) The number of spikes significantly decreased under anesthesia compared to sleep (*p* = 0.0006, Cohen's *d* = 0.6996), but no statistically significant difference was observed between anesthetic‐state and wakefulness (*p* = 0.0648, Cohen's *d* = 0.3505) or between wakefulness and sleep (*p* = 0.3654, Cohen's *d* = −0.1679). (2) The number of ripples significantly increased under anesthesia compared to wakefulness (*p* = 0.0001, Cohen's *d* = −0.8217) and were also elevated during sleep relative to wakefulness (*p* = 0.0413, Cohen's *d* = −0.3898). However, no significant difference was found between anesthetic‐state and sleep in ripple counts (*p* = 0.2094, Cohen's *d* = −0.2344). (3) The number of FRs was significantly higher under anesthesia compared to both sleep and wakefulness (sleep, *p* = 0.0001, Cohen's *d* = −0.8026; wakefulness, *p* = 0.0000, Cohen's *d* = −0.9242), while there was no statistical difference between wakefulness and sleep (*p* = 0.7806, Cohen's *d* = 0.0513) (Figure 3A, Table S3).

When comparing the changes in interictal epileptiform discharges between the SOZ and NSOZ across different states of consciousness: Compared to wakefulness, spike counts increased during sleep without significant differences between the SOZ and NSOZ (*p* = 0.1501, Cohen's *d* = 0.2699). Under anesthesia, spikes decreased more in the SOZ than in the NSOZ when compared to sleep (*p* = 0.0028, Cohen's *d* = −0.5955), but no difference was observed between the SOZ and NSOZ when compared to wakefulness (*p* = 0.0504, Cohen's *d* = −0.3727). Compared to wakefulness, HFO counts increased during sleep, but no significant difference was found between the SOZ and NSOZ (ripple, *p* = 0.1090, Cohen's *d* = 0.3019; FR, *p* = 0.0831, Cohen's *d* = 0.3277). Under anesthesia, both ripples and FRs increased compared to wakefulness and sleep, yet the extent of the increase was not significantly different between the SOZ and NSOZ (ripple, awake, *p* = 0.1516, Cohen's *d* = 0.2689; ripple, sleep, *p* = 0.2268, Cohen's *d* = −0.2255; FR, awake, *p* = 0.4058, Cohen's *d* = −0.1540; FR, sleep, *p* = 0.3854, Cohen's *d* = −0.1609) (Figure 3B, Table S4).

### 3.4 The Influence of Anesthesia on Different Cortical Regions and Age Groups

Comparisons of band energy changes across different states of consciousness revealed that energy levels in the posterior brain regions decreased under anesthesia, while those in the Rolandic area increased significantly.

**FIGURE 4:**
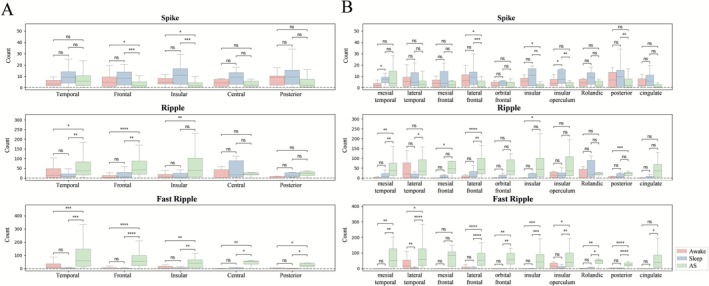
The impact of anesthesia on different brain areas (A) and cortical regions (B). *p < 0.05, **p < 0.01, ***p < 0.001, ****p < 0.0001, AS, anesthetic‐state; ns, nonsignificant.

Analysis of interictal epileptiform discharges across different brain areas showed: (1) The number of spikes did not change under anesthesia compared to wakefulness or sleep in the temporal lobe, central area, and posterior area. (2) The number of ripples remained unchanged under anesthesia compared to wakefulness or sleep in the central area and posterior area. (3) The number of FRs showed a significant increase under anesthesia across all examined brain areas (Figure 4A, Table S5).

Further analysis of interictal discharges in specific cortical regions revealed: (1) The number of spikes did not change under anesthesia compared to wakefulness or sleep in the mesial temporal cortex, lateral temporal cortex, mesial frontal cortex, orbital frontal cortex, Rolandic cortex, and cingulate cortex. (2) The number of ripples did not change under anesthesia compared to wakefulness or sleep in the orbital frontal cortex, insular operculum cortex, Rolandic cortex, and cingulate cortex. (3) The number of FRs remained unchanged under anesthesia compared to wakefulness or sleep only in the mesial frontal cortex and cingulate cortex (Figure 4B, Table S6).

**FIGURE 5:**
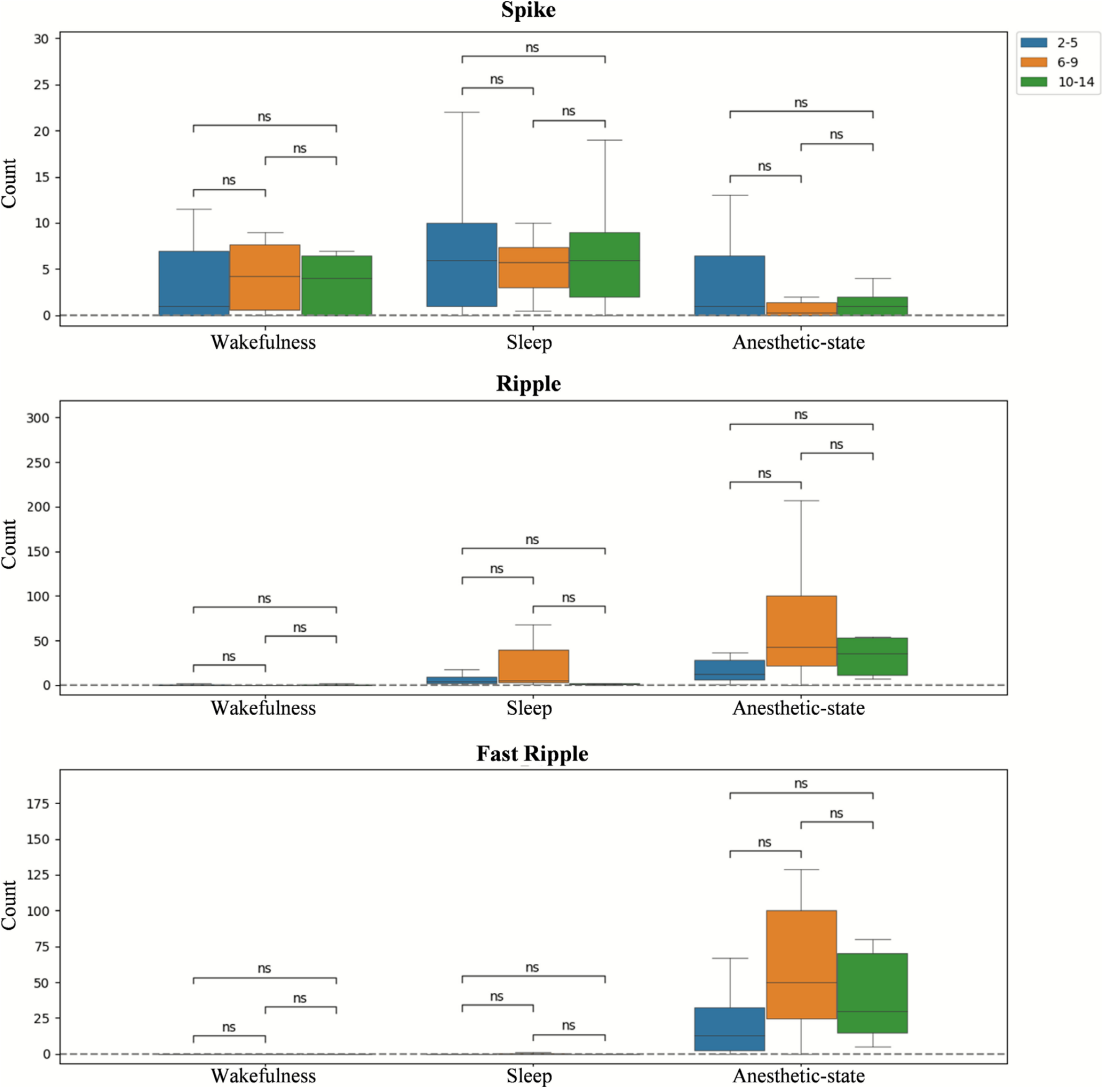
The effect of anesthesia on different age groups. ns, nonsignificant.

Regarding age groups, neither band energy analysis nor interictal epileptiform discharge counts showed statistically significant differences, though younger patients exhibited greater individual variability (Figure 5, Table S7).

## 4 Discussion

We conducted a retrospective study involving 30 patients, in which SEEG data were recorded during wakefulness, sleep, and anesthesia. The key findings include:

1. PSD generally increased under anesthesia, particularly in the high‐frequency bands. However, no statistical differences were observed in energy changes between the SOZ and NSOZ when compared to wakefulness or sleep, suggesting that the epileptogenic network remained unchanged.
2. The number of spikes decreased under anesthesia, particularly within the SOZ, while HFOs increased under anesthesia. However, the extent of the increase did not differ between the SOZ and NSOZ.
3. The number of HFOs appeared to be a more reliable interictal biomarker in ioEEG, particularly in the frontal cortex and cingulate cortex, and was not influenced by age.

These findings suggest that, under specific conditions, ioEEG may not be significantly affected by anesthetic agents when intraoperative interictal signals are used to delineate SOZ. Moreover, ioEEG could help improve epilepsy surgery outcomes by utilizing FRs as biomarkers in specific brain regions, with no age‐related limitations in pediatric patients.

Unlike intra‐operative electrocorticography, SEEG allows for the recording of electrical activity from both superficial and deeper brain structures, capturing interictal activity during wakefulness and sleep during chronic monitoring. This provides a more comprehensive assessment of the effects of anesthetic agents. In our study, ioEEG was recorded before surgery under a standardized anesthesia protocol, unlike other studies where HFO analysis in chronic or depth EEG recordings may not be confounded by anesthetic agents, which makes it difficult to draw comparisons between HFO rates in extra‐ and intra‐operative recordings. Our study design imposed no additional burden on patients, and pre‐surgical recordings after anesthesia allowed for direct comparison with ioEEG. Furthermore, automated detection methods facilitated this study, overcoming previous challenges in monitoring HFOs during ioEEG.

### 4.1 Mechanistic Insight Into Propofol's Effect on Neuronal Activity

Propofol's influence on epileptiform activity and EEG biomarkers is multifaceted and extends beyond its well‐recognized potentiation of GABAA receptor–mediated inhibition. Propofol enhances GABAergic currents by increasing receptor affinity and chloride conductance, thereby hyperpolarizing neuronal membranes and reducing overall excitability. This mechanism aligns well with our observation of spike suppression, consistent with prior reports demonstrating anesthetic‐induced attenuation of pathological discharges [12]. Interestingly, we also observed an increase in HFOs under propofol, a phenomenon that appears paradoxical given that HFOs are often associated with increased local excitability and epileptogenicity [13]. Several mechanisms may account for this counterintuitive finding.

First, regional heterogeneity in GABAA receptor subunit composition may lead to differential responses to propofol across cortical and subcortical structures. Subtypes with distinct pharmacosensitivity may produce localized disinhibition even in the context of global inhibitory tone [14]. Second, alterations in thalamocortical network dynamics induced by propofol may promote coherent high‐frequency synchronization despite overall suppression of low‐frequency excitability [15, 16]. Third, propofol may reduce inhibitory surround within epileptogenic zones—by disproportionately suppressing broad‐field inhibitory interneurons relative to locally synchronized microcircuits—thereby unmasking intrinsic high‐frequency activity [15, 17]. These mechanisms have been proposed in both basic electrophysiological and clinical EEG studies, and they provide potential explanations for the emergence of HFOs despite general suppression of spikes.

Age‐dependent neurodevelopmental physiology may further modulate the EEG response to propofol [16]. GABAergic signaling in early childhood exhibits distinct features compared with adolescents and adults [18]. In infants and younger children, relatively high expression of NKCC1 and low expression of KCC2 maintain intracellular chloride at depolarizing levels [19]. As a result, GABAergic currents can be paradoxically excitatory, potentially altering propofol's pharmacodynamic profile in the 2–5‐year age group. Even in older children, incomplete maturation of chloride transporters may shape responses to GABAergic anesthetics during cortical synchrony. Together, these developmental factors suggest that the epileptogenic network's responsiveness to propofol may differ substantially between younger (2–5 years) and older (10–14 years) patients. Although this study did not reveal statistically significant age‐related differences, our data indicate that younger patients exhibited greater individual variability, suggesting that age‐dependent factors may indeed modulate the electrophysiological response to propofol. These potential differences need further investigation in future studies.

### 4.2 The Significance of PSD in This Study

PSD reflects the distribution of EEG signal power across frequencies. Differences in PSD have been reported in temporal lobe epilepsy patients compared to healthy controls [20], and across various epilepsy syndromes, including genetic generalized epilepsy [21] and idiopathic generalized epilepsy [22]. PSD changes have also been observed during ictal periods and subclinical discharges [23] and in surgical responders to deep brain stimulation [24] and thermocoagulation [25], highlighting its potential role in characterizing the epileptogenic network and the effects of surgical interventions.

In this study, power across all frequency bands increased under anesthesia compared to wakefulness and sleep, confirming that anesthetic agents do impact brain networks. However, when analyzing the SOZ and NSOZ separately, comparisons with both wakefulness and sleep demonstrated that energy levels remained unchanged across all frequency bands under anesthesia, indicating that while anesthesia affects brain networks, it does not alter the epileptogenic network. This finding suggests that the differentiation between SOZ and NSOZ remains stable under anesthesia, reinforcing the reliability of ioEEG evaluations.

A recent study also reported that perampanel administration resulted in a mild but widespread increase in EEG theta power without affecting EEG connectivity [26]. The observed PSD changes in this study suggest that anesthetic agents may differentially affect cortical regions, decreasing activity in posterior brain areas while increasing activity in the Rolandic cortex. Given the unreliable nature of interictal discharges in these regions under anesthesia, future studies should evaluate the clinical utility of ioEEG in posterior brain areas and the Rolandic cortex.

### 4.3 Individualized Application of IoEEG

IoEEG provides neurosurgeons with a real‐time assessment of pathological neuronal activity, aiding in the delineation of the SOZ, particularly in focal and superficial lesions [27]. Additionally, it facilitates functional mapping and detection of residual epileptiform activity after the initial surgical procedure [28]. However, several challenges exist, including the short duration of intra‐operative recordings compared to chronic extra‐operative monitoring (minutes vs. days), the impact of anesthetic agents on local‐field potentials, and the potential confounding by tissue injury [29, 30]. These limitations have led to ongoing debate regarding the clinical utility of ioEEG [31]. A recent meta‐analysis reported a 74% rate of favorable seizure outcomes following ioEEG‐guided tailored resections, with significant heterogeneity [9].

Complete resection of tissue exhibiting interictal epileptiform discharges in ioEEG correlated with favorable seizure control, whereas incomplete resections were associated with a higher recurrence rate [9]. However, an excessively aggressive approach to resection—driven by interictal spikes rather than the true SOZ—may increase the risk of neurological deficits [32]. A randomized controlled trial on ioEEG demonstrated that HFOs were not superior to interictal epileptic discharges across the entire study cohort. However, after adjusting for poor pathology prognosis, HFOs demonstrated non‐inferiority in extratemporal lobe epilepsy [33]. Numerous studies have underscored the value of ioEEG in specific pathologies, including epilepsy associated with tumors [31, 34] and malformations of cortical development [8].

To our knowledge, this is the first study to assess the value of ioEEG across different cortical regions using specific interictal signals such as spikes and HFOs. Our results indicate an increase in ripples in the lateral frontal and mesial temporal cortices, while FRs increased across nearly all regions. However, no significant differences were found between the SOZ and NSOZ, suggesting that FRs may serve as reliable biomarkers to confirm the resection area, particularly in the frontal and cingulate cortices. Given the ongoing debate regarding the role of ioEEG in temporal lobe epilepsy [10, 11, 35], it is crucial to assess its accuracy in extratemporal lobe epilepsy. Our findings provide new insights into this issue, and offer region‐specific guidance for surgical interventions. As epilepsy surgery is increasingly performed in young children, where successful interventions can significantly improve social, psychological, and cognitive development [5], our findings support the use of ioEEG in pediatric epilepsy surgery without age‐related restrictions.

### 4.4 The Value of FRs in ioEEG

Postoperative ioEEG spikes may arise from surgical manipulation of the neocortex, particularly at resection margins. As a result, spike rates in resected regions have not been consistently correlated with surgical outcomes, which aligns with some previous studies [36]. Additionally, interobserver variability in spike types poses challenges for reliable interpretation of ioEEG [37]. Our findings confirmed that spike rates recorded via SEEG are likely influenced by general anesthesia, whereas HFOs are more strongly associated with the SOZ than interictal epileptic discharges such as spikes [36]. While ripples can occur physiologically in the mesiotemporal, occipital, and sensorimotor areas [38], distinguishing between physiological and pathological ripples remains challenging [39]. Previous intra‐operative studies have reported that ripple rates in resected tissue were not significantly higher than in non‐resected tissue [40, 41].

Unlike ripples, FRs are not typically observed in healthy brain tissue unless they are elicited by stimulation [42]. Furthermore, the presence of FRs in resected tissue has been associated with improved seizure outcomes, unlike spikes and ripples [36]. Additionally, automatic detection of HFOs—especially FRs—is more feasible than for spikes, facilitating real‐time surgical guidance using ioEEG [33]. Among interictal biomarkers, FRs demonstrate the highest combination of positive predictive value (100%) and negative predictive value (76%) for predicting postoperative seizures [43]. Notably, postoperative ioEEG studies suggest that FR presence is the strongest predictor of seizure recurrence, further supporting its role in surgical decision‐making [44]. Given these findings, confirming the reliability of FRs under general anesthesia is crucial for optimizing ioEEG‐guided epilepsy surgery.

### 4.5 Limitation

A limitation of our study is that it was designed to evaluate the relationship between HFOs recorded under propofol and the clinically defined SOZ, rather than to prove that intraoperative HFOs predict long‐term surgical outcome. In the present cohort, intraoperative HFOs were compared with the clinically determined SOZ and with the resection margins, but HFOs were not used prospectively to alter the surgical plan. Therefore, our data cannot directly address whether HFO‐guided resections improve long‐term outcome. We acknowledge this as an important limitation and plan to extend clinical follow‐up of this cohort and to perform a dedicated analysis relating HFO‐identified regions to the extent of resection and to long‐term seizure outcome.

## 5 Conclusion

This study investigated the impact of anesthesia with propofol on the detection and analysis of epileptic discharges using SEEG in pediatric epilepsy surgery. Our results indicate that general anesthesia significantly affects the number and frequency of epileptic discharges, particularly by reducing spikes while increasing HFOs, including ripples and FRs. Notably, HFOs—particularly in the frontal and cingulate cortices—emerged as a reliable biomarker for delineating the SOZ and guiding surgical resections. PSD analysis demonstrated an increase in high‐frequency power under anesthesia, though no significant differences were observed between the SOZ and NSOZ. Furthermore, no significant age‐related variability was identified, supporting the applicability of ioEEG in pediatric epilepsy surgery. Overall, these findings suggest that anesthesia does not significantly compromise the validity of ioEEG, particularly with respect to HFOs, which may help optimize ioEEG‐guided epilepsy surgery.

## Funding

This work was supported by “Yangfan” Program by the Beijing Municipal Hospital Administration Center (Grant No. YGLX 202531) and the National Natural Science Foundation of China (Grant No. 82301638). The authors have no personal, financial, or institutional interest in any of the drugs, materials, or devices described in this article.

## Ethics Statement

This study was approved by the Clinical Research Ethics Committee of Beijing Children's Hospital (No. IEC‐C‐006‐A04‐V.06).

## Consent

Written informed consent was obtained from all patients and/or their legal guardians.

## Conflicts of Interest

The authors declare no conflicts of interest.

## Supporting information


**Table S1:** Analysis of power spectral density (PSD) across different states of consciousness (Figure 2A).
**Table S2:** Analysis of changes of power spectral density (PSD) across EZ and NEZ (Figure 2B).
**Table S3:** Analysis of interictal epileptiform discharges across different states of consciousness (Figure 3A).
**Table S4:** Analysis of changes of interictal epileptiform discharges across EZ and NEZ (Figure 3B)
**Table S5:** Analysis of interictal epileptiform discharges across different states of consciousness in different brain areas (Figure 4A).
**Table S6:** Analysis of interictal epileptiform discharges across different states of consciousness in different cortical regions (Figure 4B).
**Table S7:** Analysis of interictal epileptiform discharges across age groups (Figure 5).

## Data Availability

The authors have nothing to report.
