# Supplementary material for: Impact of Anesthesia With Propofol on Epileptic Discharges Recorded by Stereo‐Electroencephalography in Pediatric Epilepsy Surgery
Source: CNS Neurosci Ther. 2026 Jan 27;32(1):e70767. doi: 10.1002/cns.70767 (PMC12835890; doi:10.1002/cns.70767)
Supplement: Supplementary file 1 — Table S1: Analysis of power spectral density (PSD) across different states of consciousness (Figure 2A). Table S2: Analysis of changes of power spectral density (PSD) across EZ and NEZ (Figure 2B). Table S3: Analysis of interictal epileptiform discharges across different states of consciousness (Figure 3A). Table S4: Analysis of changes of interictal epileptiform discharges across EZ and NEZ (Figure 3B) Table S5: Analysis of interictal epileptiform discharges across different states of consciousness in different brain areas (Figure 4A). Table S6: Analysis of interictal epileptiform discharges across different states of consciousness in different cortical regions (Figure 4B). Table S7: Analysis of interictal epileptiform discharges across age groups (Figure 5). [file CNS-32-e70767-s001.docx]

Table S1. Analysis of power spectral density (PSD) across different states of consciousness (Figure 2A)

| States | Frequency | t | p | Cohen’s d |
| --- | --- | --- | --- | --- |
| Awake vs Sleep | delta | -51.0525 | 0.0000 | -0.8899 |
|  | theta | -27.8420 | 0.0000 | -0.4853 |
|  | alpha | -24.3905 | 0.0000 | -0.4252 |
|  | beta | -0.3831 | 0.7017 | -0.0067 |
|  | gamma | 8.9992 | 0.0000 | 0.1569 |
|  | high_gamma | 13.7391 | 0.0000 | 0.2395 |
|  | ripple | 14.2249 | 0.0000 | 0.2480 |
| Awake vs AS | delta | -44.9197 | 0.0000 | -0.7830 |
|  | theta | -40.1891 | 0.0000 | -0.7006 |
|  | alpha | -52.6495 | 0.0000 | -0.9178 |
|  | beta | -54.6743 | 0.0000 | -0.9531 |
|  | gamma | -60.8275 | 0.0000 | -1.0603 |
|  | high_gamma | -63.4304 | 0.0000 | -1.1057 |
|  | ripple | -63.4742 | 0.0000 | -1.1065 |
| Sleep vs AS | delta | -29.7076 | 0.0000 | -0.5178 |
|  | theta | -36.0435 | 0.0000 | -0.6283 |
|  | alpha | -50.7882 | 0.0000 | -0.8853 |
|  | beta | -58.3711 | 0.0000 | -1.0175 |
|  | gamma | -68.0150 | 0.0000 | -1.1856 |
|  | high_gamma | -72.6702 | 0.0000 | -1.2668 |
|  | ripple | -71.7684 | 0.0000 | -1.2510 |

Table S2. Analysis of changes of power spectral density (PSD) across EZ and NEZ (Figure 2B)

| States | Frequency | t | p | Cohen’s d |
| --- | --- | --- | --- | --- |
| Sleep - Awake | delta | -0.8007 | 0.4298 | -0.1462 |
|  | theta | 0.2055 | 0.8386 | 0.0375 |
|  | alpha | 0.0766 | 0.9395 | 0.0140 |
|  | beta | 0.2337 | 0.8169 | 0.0427 |
|  | gamma | 0.6203 | 0.5399 | 0.1132 |
|  | high_gamma | 0.5290 | 0.6008 | 0.0966 |
|  | ripple | 0.8291 | 0.4138 | 0.1514 |
| AS - awake | delta | -1.0334 | 0.3099 | -0.1887 |
|  | theta | 0.1019 | 0.9196 | 0.0186 |
|  | alpha | -0.0399 | 0.9684 | -0.0073 |
|  | beta | -0.1714 | 0.8651 | -0.0313 |
|  | gamma | 1.7292 | 0.0944 | 0.3157 |
|  | high_gamma | 1.4247 | 0.1649 | 0.2601 |
|  | ripple | 1.5644 | 0.1286 | 0.2856 |
| AS - Sleep | delta | -0.6760 | 0.5044 | -0.1234 |
|  | theta | -0.0035 | 0.9972 | -0.0006 |
|  | alpha | -0.0978 | 0.9228 | -0.0179 |
|  | beta | -0.2941 | 0.7708 | -0.0537 |
|  | gamma | 1.4770 | 0.1505 | 0.2697 |
|  | high_gamma | 1.2374 | 0.2259 | 0.2259 |
|  | ripple | 1.2717 | 0.2136 | 0.2322 |

Table S3. Analysis of interictal epileptiform discharges across different states of consciousness (Figure 3A)

| IID | States | t | p | Cohen’s d |
| --- | --- | --- | --- | --- |
| Spike | Awake vs Sleep | -0.9195 | 0.3654 | -0.1679 |
|  | Awake vs AS | 1.9195 | 0.0648 | 0.3505 |
|  | Sleep vs AS | 3.8320 | 0.0006 | 0.6996 |
| Ripple | Awake vs Sleep | -2.1351 | 0.0413 | -0.3898 |
|  | Awake vs AS | -4.5006 | 0.0001 | -0.8217 |
|  | Sleep vs AS | -1.2837 | 0.2094 | -0.2344 |
| Fast Ripple | Awake vs Sleep | 0.2811 | 0.7806 | 0.0513 |
|  | Awake vs AS | -5.0622 | 0.0000 | -0.9242 |
|  | Sleep vs AS | -4.3958 | 0.0001 | -0.8026 |

Table S4. Analysis of changes of interictal epileptiform discharges across EZ and NEZ (Figure 3B)

| IID | States | t | p | Cohen’s d |
| --- | --- | --- | --- | --- |
| Spike | Sleep - Awake | 1.4782 | 0.1501 | 0.2699 |
|  | AS - Awake | -2.0415 | 0.0504 | -0.3727 |
|  | AS - Sleep | -3.2617 | 0.0028 | -0.5955 |
| Ripple | Sleep - Awake | 1.6538 | 0.1090 | 0.3019 |
|  | AS - Awake | 1.4728 | 0.1516 | 0.2689 |
|  | AS - Sleep | -1.2350 | 0.2268 | -0.2255 |
| Fast Ripple | Sleep - Awake | 1.7951 | 0.0831 | 0.3277 |
|  | AS - Awake | -0.8437 | 0.4058 | -0.1540 |
|  | AS - Sleep | -0.8813 | 0.3854 | -0.1609 |

Table S5. Analysis of interictal epileptiform discharges across different states of consciousness in different brain areas (Figure 4A)

| IID | Area | Stage1 | Stage2 | t | p | Cohen’s d |
| --- | --- | --- | --- | --- | --- | --- |
| Spike | Temporal | Awake | Sleep | -3.9685 | 0.0006 | -0.7937 |
|  |  | Awake | AS | -0.7208 | 0.4780 | -0.1442 |
|  |  | Sleep | AS | 1.3113 | 0.2022 | 0.2623 |
|  | Frontal | Awake | Sleep | -0.7497 | 0.4597 | -0.1392 |
|  |  | Awake | AS | 1.9509 | 0.0611 | 0.3623 |
|  |  | Sleep | AS | 3.2628 | 0.0029 | 0.6059 |
|  | Insular | Awake | Sleep | -1.3220 | 0.1992 | -0.2699 |
|  |  | Awake | AS | 1.9978 | 0.0577 | 0.4078 |
|  |  | Sleep | AS | 3.6100 | 0.0015 | 0.7369 |
|  | Central | Awake | Sleep | 0.2426 | 0.8180 | 0.0990 |
|  |  | Awake | AS | 0.9207 | 0.3994 | 0.3759 |
|  |  | Sleep | AS | 1.6212 | 0.1659 | 0.6619 |
|  | Posterior | Awake | Sleep | -0.8981 | 0.3925 | -0.2840 |
|  |  | Awake | AS | 0.4573 | 0.6583 | 0.1446 |
|  |  | Sleep | AS | 1.6113 | 0.1416 | 0.5095 |
| Ripple | Temporal | Awake | Sleep | 1.7782 | 0.0880 | 0.3556 |
|  |  | Awake | AS | -2.5126 | 0.0191 | -0.5025 |
|  |  | Sleep | AS | -3.0815 | 0.0051 | -0.6163 |
|  | Frontal | Awake | Sleep | -1.9697 | 0.0588 | -0.3658 |
|  |  | Awake | AS | -5.3366 | 0.0000 | -0.9910 |
|  |  | Sleep | AS | -3.0434 | 0.0050 | -0.5651 |
|  | Insular | Awake | Sleep | -1.1250 | 0.2722 | -0.2296 |
|  |  | Awake | AS | -3.2600 | 0.0034 | -0.6654 |
|  |  | Sleep | AS | -2.0357 | 0.0535 | -0.4155 |
|  | Central | Awake | Sleep | -1.7869 | 0.1340 | -0.7295 |
|  |  | Awake | AS | 0.2091 | 0.8426 | 0.0854 |
|  |  | Sleep | AS | 1.2968 | 0.2513 | 0.5294 |
|  | Posterior | Awake | Sleep | -1.2319 | 0.2492 | -0.3896 |
|  |  | Awake | AS | -1.6226 | 0.1391 | -0.5131 |
|  |  | Sleep | AS | 0.4625 | 0.6547 | 0.1462 |
| Fast Ripple | Temporal | Awake | Sleep | 1.2555 | 0.2214 | 0.2511 |
|  |  | Awake | AS | -3.4551 | 0.0021 | -0.6910 |
|  |  | Sleep | AS | -4.8910 | 0.0001 | -0.9782 |
|  | Frontal | Awake | Sleep | 0.9163 | 0.3674 | 0.1701 |
|  |  | Awake | AS | -4.9111 | 0.0000 | -0.9120 |
|  |  | Sleep | AS | -4.8970 | 0.0000 | -0.9094 |
|  | Insular | Awake | Sleep | 1.0696 | 0.2959 | 0.2183 |
|  |  | Awake | AS | -3.5055 | 0.0019 | -0.7156 |
|  |  | Sleep | AS | -3.5306 | 0.0018 | -0.7207 |
|  | Central | Awake | Sleep | -1.9575 | 0.1076 | -0.7991 |
|  |  | Awake | AS | -4.0506 | 0.0098 | -1.6537 |
|  |  | Sleep | AS | -3.6642 | 0.0145 | -1.4959 |
|  | Posterior | Awake | Sleep | 0.4863 | 0.6384 | 0.1538 |
|  |  | Awake | AS | -2.1914 | 0.0561 | -0.6930 |
|  |  | Sleep | AS | -2.3377 | 0.0442 | -0.7392 |

Tabel S6. Analysis of interictal epileptiform discharges across different states of consciousness in different cortical regions (Figure 4B)

| IID | Regions | Stage1 | Stage2 | t | p | Cohen’s d |
| --- | --- | --- | --- | --- | --- | --- |
| Spike | Mesial Temporal | Awake | Sleep | -3.6712 | 0.0012 | -0.7342 |
|  |  | Awake | AS | -2.0517 | 0.0513 | -0.4103 |
|  |  | Sleep | AS | -0.1092 | 0.9140 | -0.0218 |
|  | Lateral Temporal | Awake | Sleep | -1.6258 | 0.1182 | -0.3390 |
|  |  | Awake | AS | 1.6520 | 0.1127 | 0.3445 |
|  |  | Sleep | AS | 2.2406 | 0.0355 | 0.4672 |
|  | Mesial Frontal | Awake | Sleep | -0.7410 | 0.4799 | -0.2470 |
|  |  | Awake | AS | 0.6348 | 0.5433 | 0.2116 |
|  |  | Sleep | AS | 1.0580 | 0.3210 | 0.3527 |
|  | Lateral Frontal | Awake | Sleep | -0.4920 | 0.6268 | -0.0947 |
|  |  | Awake | AS | 2.2925 | 0.0302 | 0.4412 |
|  |  | Sleep | AS | 3.8397 | 0.0007 | 0.7389 |
|  | Orbital Frontal | Awake | Sleep | -0.5434 | 0.5935 | -0.1247 |
|  |  | Awake | AS | -0.2515 | 0.8043 | -0.0577 |
|  |  | Sleep | AS | 0.3407 | 0.7373 | 0.0782 |
|  | Insular | Awake | Sleep | -1.2645 | 0.2193 | -0.2637 |
|  |  | Awake | AS | 2.2419 | 0.0354 | 0.4675 |
|  |  | Sleep | AS | 2.9538 | 0.0073 | 0.6159 |
|  | Insular Operculum | Awake | Sleep | -2.1487 | 0.0455 | -0.4929 |
|  |  | Awake | AS | 0.9953 | 0.3328 | 0.2283 |
|  |  | Sleep | AS | 2.8106 | 0.0116 | 0.6448 |
|  | Rolandic | Awake | Sleep | 0.2426 | 0.8180 | 0.0990 |
|  |  | Awake | AS | 0.9207 | 0.3994 | 0.3759 |
|  |  | Sleep | AS | 1.6212 | 0.1659 | 0.6619 |
|  | Posterior | Awake | Sleep | -1.0651 | 0.3146 | -0.3368 |
|  |  | Awake | AS | 0.2890 | 0.7791 | 0.0914 |
|  |  | Sleep | AS | 1.4730 | 0.1748 | 0.4658 |
|  | Cingulate | Awake | Sleep | -0.7874 | 0.4391 | -0.1607 |
|  |  | Awake | AS | 1.7066 | 0.1014 | 0.3484 |
|  |  | Sleep | AS | 2.9756 | 0.0068 | 0.6074 |
| Ripple | Mesial Temporal | Awake | Sleep | -0.8461 | 0.4058 | -0.1692 |
|  |  | Awake | AS | -3.0851 | 0.0051 | -0.6170 |
|  |  | Sleep | AS | -2.9603 | 0.0068 | -0.5921 |
|  | Lateral Temporal | Awake | Sleep | 3.2527 | 0.0036 | 0.6782 |
|  |  | Awake | AS | -1.0256 | 0.3162 | -0.2139 |
|  |  | Sleep | AS | -2.7807 | 0.0109 | -0.5798 |
|  | Mesial Frontal | Awake | Sleep | -1.2112 | 0.2604 | -0.4037 |
|  |  | Awake | AS | -2.0898 | 0.0700 | -0.6966 |
|  |  | Sleep | AS | -1.1659 | 0.2772 | -0.3886 |
|  | Lateral Frontal | Awake | Sleep | -1.4571 | 0.1571 | -0.2804 |
|  |  | Awake | AS | -4.5705 | 0.0001 | -0.8796 |
|  |  | Sleep | AS | -3.7874 | 0.0008 | -0.7289 |
|  | Orbital Frontal | Awake | Sleep | -0.7479 | 0.4642 | -0.1716 |
|  |  | Awake | AS | -2.5179 | 0.0215 | -0.5777 |
|  |  | Sleep | AS | -1.9570 | 0.0660 | -0.4490 |
|  | Insular | Awake | Sleep | -2.1593 | 0.0420 | -0.4502 |
|  |  | Awake | AS | -3.2540 | 0.0036 | -0.6785 |
|  |  | Sleep | AS | -1.9926 | 0.0589 | -0.4155 |
|  | Insular Operculum | Awake | Sleep | 0.0135 | 0.9894 | 0.0031 |
|  |  | Awake | AS | -1.7645 | 0.0946 | -0.4048 |
|  |  | Sleep | AS | -1.8676 | 0.0782 | -0.4285 |
|  | Rolandic | Awake | Sleep | -1.7869 | 0.1340 | -0.7295 |
|  |  | Awake | AS | 0.2091 | 0.8426 | 0.0854 |
|  |  | Sleep | AS | 1.2968 | 0.2513 | 0.5294 |
|  | Posterior | Awake | Sleep | -1.0478 | 0.3220 | -0.3314 |
|  |  | Awake | AS | -1.6265 | 0.1383 | -0.5143 |
|  |  | Sleep | AS | 0.1413 | 0.8907 | 0.0447 |
|  | Cingulate | Awake | Sleep | -1.6728 | 0.1079 | -0.3415 |
|  |  | Awake | AS | -3.8136 | 0.0009 | -0.7784 |
|  |  | Sleep | AS | -1.0600 | 0.3002 | -0.2164 |
| Fast Ripple | Mesial Temporal | Awake | Sleep | -0.4969 | 0.6238 | -0.0994 |
|  |  | Awake | AS | -3.2177 | 0.0037 | -0.6435 |
|  |  | Sleep | AS | -3.6838 | 0.0012 | -0.7368 |
|  | Lateral Temporal | Awake | Sleep | 3.0376 | 0.0060 | 0.6334 |
|  |  | Awake | AS | -2.1704 | 0.0411 | -0.4525 |
|  |  | Sleep | AS | -4.4768 | 0.0002 | -0.9335 |
|  | Mesial Frontal | Awake | Sleep | -1.0908 | 0.3071 | -0.3636 |
|  |  | Awake | AS | -0.9014 | 0.3937 | -0.3005 |
|  |  | Sleep | AS | -0.8293 | 0.4310 | -0.2764 |
|  | Lateral Frontal | Awake | Sleep | 1.0111 | 0.3213 | 0.1946 |
|  |  | Awake | AS | -4.5182 | 0.0001 | -0.8695 |
|  |  | Sleep | AS | -4.6286 | 0.0001 | -0.8908 |
|  | Orbital Frontal | Awake | Sleep | -1.3867 | 0.1825 | -0.3181 |
|  |  | Awake | AS | -3.5305 | 0.0024 | -0.8100 |
|  |  | Sleep | AS | -3.4373 | 0.0029 | -0.7886 |
|  | Insular | Awake | Sleep | -0.8967 | 0.3796 | -0.1870 |
|  |  | Awake | AS | -3.8939 | 0.0008 | -0.8119 |
|  |  | Sleep | AS | -3.8040 | 0.0010 | -0.7932 |
|  | Insular Operculum | Awake | Sleep | 1.2482 | 0.2279 | 0.2864 |
|  |  | Awake | AS | -2.8511 | 0.0106 | -0.6541 |
|  |  | Sleep | AS | -3.1129 | 0.0060 | -0.7141 |
|  | Rolandic | Awake | Sleep | -1.9575 | 0.1076 | -0.7991 |
|  |  | Awake | AS | -4.0506 | 0.0098 | -1.6537 |
|  |  | Sleep | AS | -3.6642 | 0.0145 | -1.4959 |
|  | Posterior | Awake | Sleep | 0.6735 | 0.5176 | 0.2130 |
|  |  | Awake | AS | -2.0550 | 0.0700 | -0.6499 |
|  |  | Sleep | AS | -2.2406 | 0.0518 | -0.7085 |
|  | Cingulate | Awake | Sleep | -0.9155 | 0.3694 | -0.1869 |
|  |  | Awake | AS | -4.3396 | 0.0002 | -0.8858 |
|  |  | Sleep | AS | -4.1959 | 0.0003 | -0.8565 |

Table S7. Analysis of interictal epileptiform discharges across age groups (Figure 5)

| IID | Stage | Age Group1 | Age Group2 | t | p | Cohen’s d |
| --- | --- | --- | --- | --- | --- | --- |
| Spike | Awake | 2-5 | 6-9 | -0.8631 | 0.4030 | -0.3875 |
|  |  | 6-9 | 10-14 | 0.4429 | 0.6641 | 0.1984 |
|  |  | 2-5 | 10-14 | -0.5395 | 0.5968 | -0.2454 |
|  | Sleep | 2-5 | 6-9 | 0.2373 | 0.8151 | 0.1023 |
|  |  | 6-9 | 10-14 | 0.0554 | 0.9565 | 0.0255 |
|  |  | 2-5 | 10-14 | 0.2777 | 0.7844 | 0.1219 |
|  | AS | 2-5 | 6-9 | 1.7192 | 0.1098 | 0.7226 |
|  |  | 6-9 | 10-14 | -0.6447 | 0.5278 | -0.2945 |
|  |  | 2-5 | 10-14 | 1.4165 | 0.1812 | 0.5844 |
| Ripple | Awake | 2-5 | 6-9 | -0.9337 | 0.3748 | -0.4289 |
|  |  | 6-9 | 10-14 | 0.9633 | 0.3605 | 0.4187 |
|  |  | 2-5 | 10-14 | 0.6236 | 0.5429 | 0.2606 |
|  | Sleep | 2-5 | 6-9 | -1.2512 | 0.2423 | -0.5747 |
|  |  | 6-9 | 10-14 | 1.3026 | 0.2249 | 0.5663 |
|  |  | 2-5 | 10-14 | 0.6994 | 0.4937 | 0.3147 |
|  | AS | 2-5 | 6-9 | -1.8500 | 0.0873 | -0.8332 |
|  |  | 6-9 | 10-14 | 1.1480 | 0.2700 | 0.5118 |
|  |  | 2-5 | 10-14 | -0.9715 | 0.3453 | -0.4408 |
| Fast Ripple | Awake | 2-5 | 6-9 | -0.0673 | 0.9471 | -0.0295 |
|  |  | 6-9 | 10-14 | -0.5016 | 0.6257 | -0.2387 |
|  |  | 2-5 | 10-14 | -0.5469 | 0.5957 | -0.2637 |
|  | Sleep | 2-5 | 6-9 | -0.9806 | 0.3413 | -0.4356 |
|  |  | 6-9 | 10-14 | 0.7966 | 0.4369 | 0.3612 |
|  |  | 2-5 | 10-14 | -0.1407 | 0.8898 | -0.0639 |
|  | AS | 2-5 | 6-9 | -1.7461 | 0.1060 | -0.7890 |
|  |  | 6-9 | 10-14 | 0.6335 | 0.5349 | 0.2875 |
|  |  | 2-5 | 10-14 | -1.1317 | 0.2800 | -0.5379 |
